# Biosecurity Primitive: Polymerase X‐based Genetic Physical Unclonable Functions

**DOI:** 10.1002/advs.202415820

**Published:** 2025-06-09

**Authors:** Zikun Zhou, Taek Kang, Jie Chen, Yesh Doctor, Jocelyn G Camposagrado, Yiorgos Makris, Alexander Pertsemlidis, Leonidas Bleris

**Affiliations:** ^1^ Bioengineering Department The University of Texas at Dallas Richardson Texas 75080 USA; ^2^ Center for Systems Biology The University of Texas at Dallas Richardson Texas 75080 USA; ^3^ Department of Biological Sciences The University of Texas at Dallas Richardson Texas 75080 USA; ^4^ Department of Electrical and Computer Engineering University of Texas at Dallas Richardson Texas 75080 USA; ^5^ Department of Pediatrics The University of Texas Health Science Center at San Antonio San Antonio Texas 78229 USA; ^6^ Department of Cell Systems & Anatomy The University of Texas Health Science Center at San Antonio San Antonio Texas 78229 USA; ^7^ Greehey Children's Cancer Research Institute The University of Texas Health Science Center at San Antonio San Antonio Texas 78229 USA

**Keywords:** barcoding, biosecurity, genetic PUFs, genome editing, PolyX

## Abstract

A Physical Unclonable Function (PUF) is a security primitive that exploits inherent variations in manufacturing protocols to generate unique, random‐like identifiers. These identifiers are used for authentication and encryption purposes in hardware security applications in the semiconductor industry. Inspired by the success of silicon PUFs, herein it is leverage Terminal deoxynucleotidyl Transferase (TdT), a template‐independent polymerase belonging to the X‐family of DNA polymerases, to augment the intrinsic entropy generated during DNA lesion repair and rapidly produce genetic PUFs that satisfy the following properties: robustness (i.e., they repeatedly produce the same output), uniqueness (i.e., they do not coincide with any other identically produced PUF), and unclonability (i.e., they are virtually impossible to replicate). Furthermore, a post‐sequencing feature selection methodology based on logistic regression to facilitate PUF classification is developed. This experimental and computational pipeline drastically reduces production time and cost compared to conventional genetic barcoding without compromising the stringent PUF criteria of uniqueness and unclonability. This results provide novel insights into the function of TdT and represent a major step toward utilization of PUFs as a biosecurity primitive for cell line authentication and provenance attestation.

## Graphical abstract

What if every cell line can carry an unforgeable genomic fingerprint—one that proves its origin, ensures its authenticity, and thwarts unauthorized duplication? Here, CRISPR‐induced DNA lesions paired with TdT‐mediated repair produce robust, unique, and unclonable genetic signatures—advancing the concept of molecular Physical Unclonable Functions (PUF) and opening new avenues for biosecurity applications in cell line authentication.

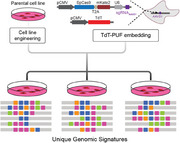

## 1 Introduction

A physical unclonable function (PUF)^[^ ^1^, ^2^, ^3^ ^]^ is a hardware security measure that enables the unique identification and authentication of a device. Silicon PUFs exploit the inherent randomness of semiconductor manufacturing processes and are innately unique and irreproducible. While these variations are random, they can be exploited to generate a unique “response” to a specific input, known as a “challenge”, based on the unique physical characteristics of each chip. Challenge‐response pairs (CRPs) serve as a basis for verification. When the authenticity of the product is to be evaluated, the product's response to a challenge is compared against a stored reference. The standard metrics for evaluating a PUF include robustness (i.e., the ability to produce consistent responses to the same challenges) and uniqueness (i.e., the ability to produce mappings that no other identically manufactured PUF can replicate). We previously introduced the first generation of genetic PUF.^[^ ^4^ ^]^ By employing amplicon sequencing of a predefined engineered genomic locus, a cell line owner can compare the resulting distinctive (nucleotide frequency) signatures against a database for verification. Drawing parallels to the utilization of silicon PUFs, this methodology confirms the bona fide procurement of the cell line, concurrently assuring the end‐user of both the provenance and the authenticity of the biological product.

To develop PUFs, we leverage genome engineering using Clustered Regularly Interspaced Short Palindromic Repeats (CRISPR).^[^ ^5^, ^6^, ^7^ ^]^ CRISPR is an immune response mechanism^[^ ^8^ ^]^ against bacteriophage infections in bacteria and archaea that has revolutionized the field of genome editing.^[^ ^9^, ^10^, ^11^, ^12^, ^13^, ^14^ ^]^ We hypothesized that a stochastic non‐homologous end joining repair, combined with molecular barcoding, can yield genetic changes that satisfy all PUF conditions of robustness, uniqueness, and unclonability. For the first stage of the protocol, we integrated a 5‐nucleotide barcode library into the *AAVS1* locus of human HEK293 cells using CRISPR‐mediated homologous recombination (HR). We then transiently transfected the barcoded cell line with a sgRNA targeting a locus adjacent to the barcodes to induce non‐homologous end‐joining repair (NHEJR). Finally, we amplified this region via PCR, sequenced the amplicons by NGS, associated the observed indels with their corresponding barcodes from the same reads, and produced a matrix of frequencies of barcode‐indel combinations.^[^ ^4^ ^]^ The (cropped) matrix of the most frequently detected barcode and indel sequences satisfies the PUF metrics of robustness, uniqueness, and unclonability.^[^ ^4^ ^]^

**Figure 1:**
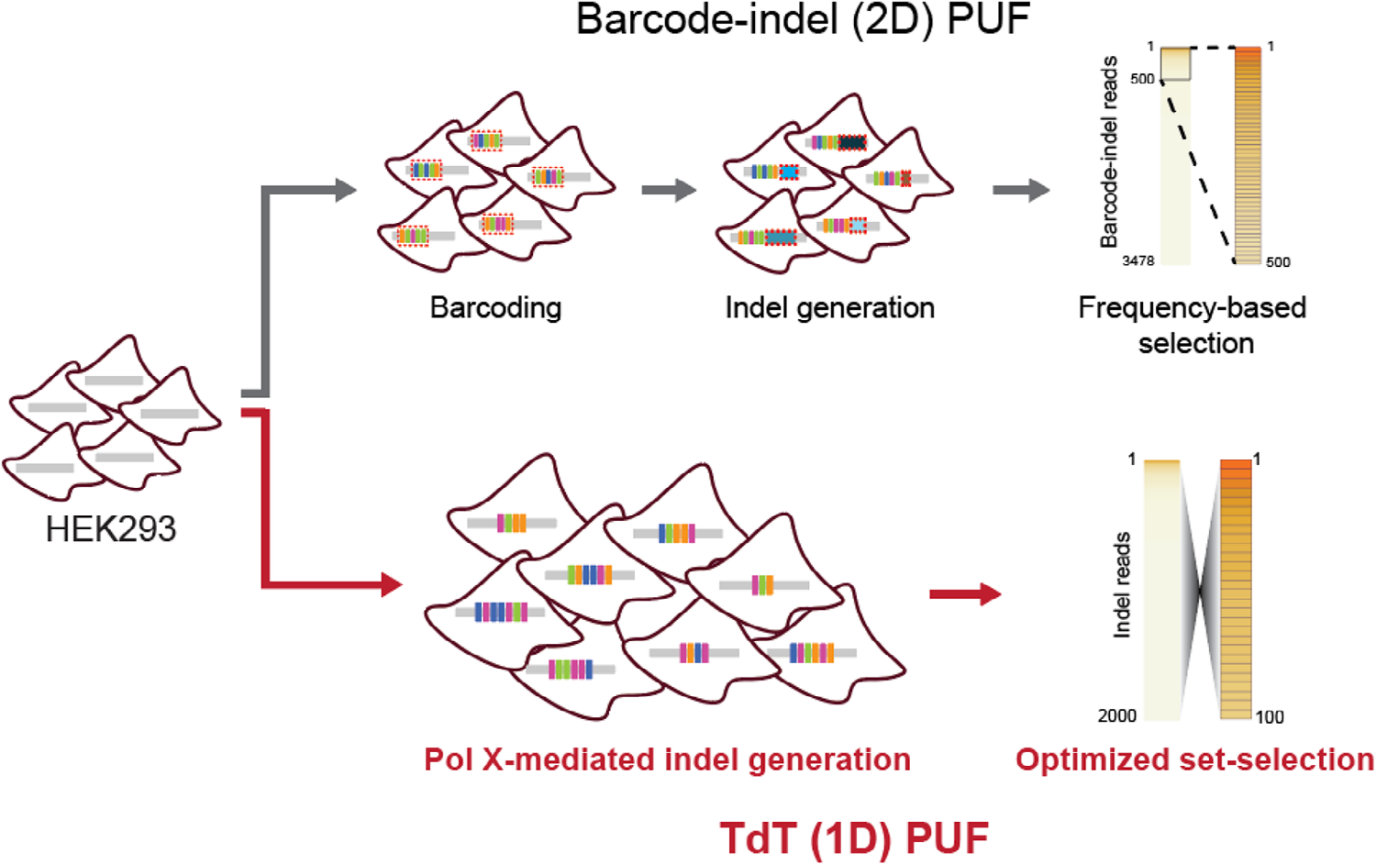
PUF engineering methodology. Overview of barcode‐indel PUFs, Cas9‐induced NHEJ indel generation and frequency‐based selection for signature verification. Compared against TdT PUF consisting of a single Cas9‐induced NHEJ indel generation and Pol X enzyme (TdT) for entropy increase, alongside optimized feature selection techniques for signature verification.

The integrity of genetic PUFs hinges on the generation of unpredictable insertions and deletions (indels) following NHEJ repair of the CRISPR‐mediated cleavage. However, as the NHEJR indels are dominated by deletions and thus do not always generate abundant genetic variability (i.e., entropy) among cell populations, our first generation of genetic PUFs relied on the random association of the indels with genetic barcodes in individual cells (**Figure** **1**). Here, we focus on the NHEJ repair pathway to prioritize random insertions and subsequently increase sequence entropy at the target locus, thereby removing the need for barcoding (Figure 1). One step in NHEJ repair involves the recruitment of DNA repair machinery to catalyze DNA end‐ and gap‐filling processes. In particular, the polymerase‐X (Pol X) family of proteins catalyze these processes in both template‐dependent and ‐independent manner, making the process error‐prone.^[^ ^15^, ^16^ ^]^ The family includes Terminal deoxynucleotidyl transferase (TdT), Pol λ, Pol µ, Pol β, and Pol σ, each with distinct substrate preferences and physiological roles.^[^ ^17^ ^]^ Among these, TdT is uniquely known to catalyze the addition of nucleotides to the 3′ ends of DNA strands, thus favoring random insertions during NHEJ.^[^ ^18^, ^19^, ^20^, ^21^ ^]^ We therefore hypothesized that by overexpressing TdT, we can direct the repair outcome toward frequent random insertions. These TdT‐mediated insertions inherently produce higher entropy than deletions and therefore represent a potential strategy to bypass the need for barcoding in PUF production. Furthermore, we develop post‐sequencing heuristic and multiclass logistic regression methodologies to leverage indels that are most informative for PUF classification.

In this study, we present a novel approach to rapidly generate genetic PUFs by harnessing the inherent repair entropy introduced by the overexpression of TdT during CRISPR‐mediated non‐homologous end joining. By biasing the DNA repair process toward frequent random insertions, we significantly increase the sequence entropy at the target locus, effectively eliminating the previous requirement for molecular barcoding. Our work provides valuable new insights into the functional characteristics of TdT and represents a significant step toward the practical implementation of genetic PUFs in commercial applications. The ability to rapidly and reliably generate PUFs within genomic safe harbors opens new avenues for secure cell line authentication and provenance verification, with far‐reaching implications for the biotechnology and pharmaceutical industries.

## 2 Results

### 2.1 Characterization of TdT‐Mediated Indels

**Figure 2:**
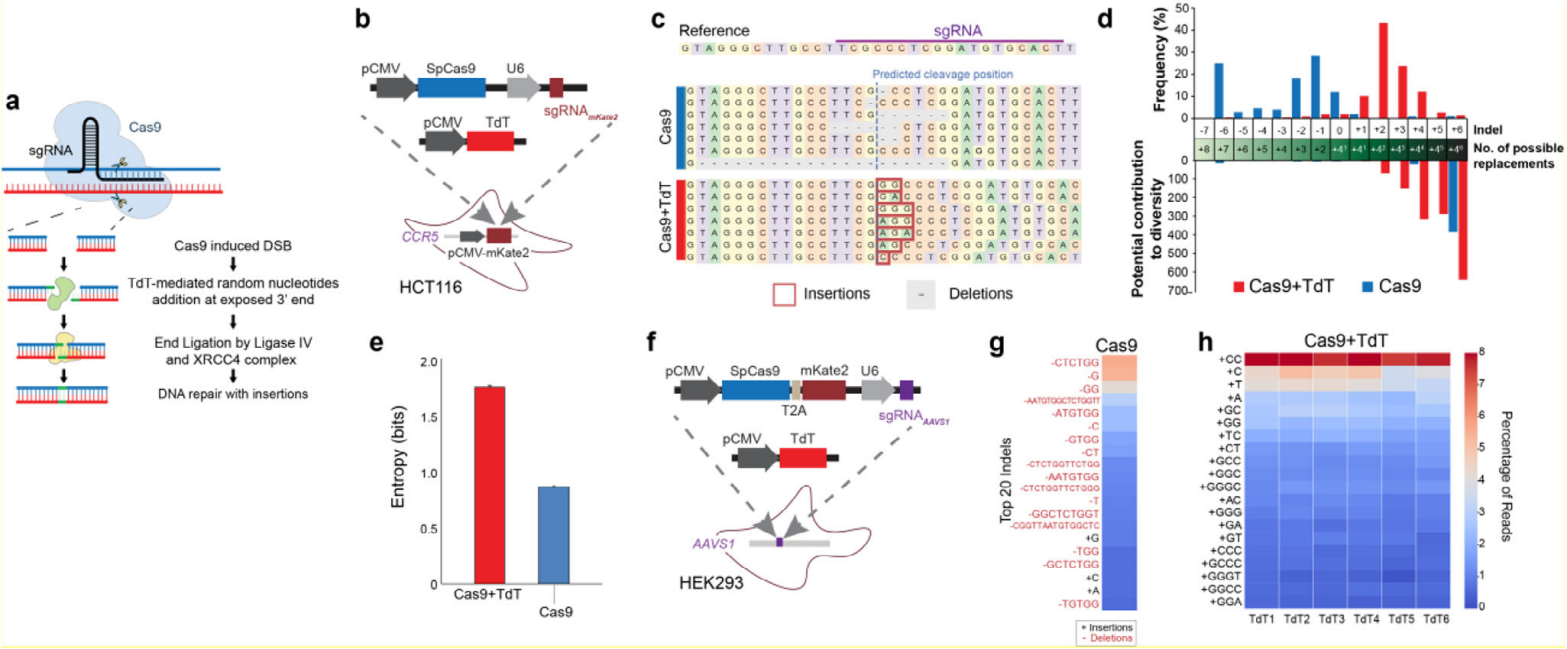
Characterization of TdT‐mediated indels. a) Illustration of the hypothesized mechanisms involved in TdT‐induced insertion‐biased NHEJ repair. b) Plasmid constructs used to target the integrated mKate2. c) Tables of the seven most frequent indels of Cas9 alone and Cas9 with TdT, generated by CRISPResso2. d) Bar graph (top) comparing indel size distributions between Cas9 alone and Cas9 alongside TdT. Bar graph (bottom) showing the expected contribution to diversity. e) Comparison between numbers of possible indels and observed indels in Cas9 alone and Cas9 with TdT samples. f) Plasmid constructs used to target the *AAVS1* site. g,h) Frequency plots of the top 20 indels at the *AAVS1* site with Cas9 g) and with Cas9‐TdT h).

The Pol X family member TdT is known to catalyze the addition of nucleotides to the 3′ ends of DNA strands (**Figure** **2**a).^[^ ^18^, ^19^, ^20^, ^21^ ^]^ We hypothesized that delivering TdT and Cas9 together would yield significant differences in the resulting indel landscape compared to the nuclease alone. We co‐transfected pCMV‐SpCas9‐U6‐sgRNA_mKate2_ (pl‐Cas9) with and without pCMV‐TdT (pl‐TdT) plasmids into HCT116 cells that harbor a pCMV‐mKate2 cassette at the CCR5 locus (Supporting Material, Plasmids). The guide RNA targets the open reading frame of mKate2 (Figure 2b). 72 h post‐transfection, we harvested genomic DNA, amplified the target locus via PCR (Supporting Material, Primers), and performed next‐generation amplicon sequencing.

To assess possible off‐target effects of the CRISPR‐Cas9 system, we also conducted in parallel T7 endonuclease I‐based mutation detection assay of top predicted off‐targets identified by CasOFFinder^[^ ^22^ ^]^ (Figure S1, Supporting Information). No cleavage was observed in these samples, indicating no detectable off‐target effects. In addition, we examined possible cytotoxic effects of TdT transfection via Annexin V/PI‐based apoptosis assay and confirmed that moderate TdT overexpression used to generate PUFs does not substantially affect cell morphology or compromise viability (Figures S2 and S3, Supporting Information).

The sequencing results were then analyzed using CRISPResso2^[^ ^23^ ^]^ to showcase the diversity of TdT‐induced indel profiles (Figure 2c). CRISPR/SpCas9 alone predominantly induced deletions (≈84% of the resulting genetic alterations), with an indel distribution enriched for deletions of one or two base pairs. The addition of pl‐TdT significantly altered this distribution, with 80% of the events being insertions, most of which were one to four base pairs in length. To illustrate the impact of insertion‐dominant repair pathway on the entropy, we calculated the theoretical expected contribution to diversity for each indel profile by multiplying the frequencies of indel size by the number of possible replacements (Figure 2d). To quantify the impact of TdT‐mediated insertions on the entropy of the edited locus, we computed Shannon entropy directly, following previous studies that used this metric to characterize the CRISPR‐induced mutation profiles.^[^ ^24^, ^25^ ^]^ To account for the difference in the number of reads between each sample, we performed a resampling procedure wherein we selected 100000 indel reads without replacement, repeated this procedure 100 times to calculate the mean Shannon entropy of the subsamples. The results show that addition of pl‐TdT nearly doubles the mean entropy from 0.86 to 1.74 bits at the target locus (Figure 2e). Moreover, the subsampled population with pl‐TdT contains a larger variety of unique indels and a pronounced shift toward insertions (Figure S4, Supporting Information). Collectively, our results demonstrate that following a SpCas9‐induced double‐strand break, TdT can introduce random nucleotides at the exposed double‐strand break during NHEJ‐mediated repair and increase the indel complexity.

To further characterize TdT operation, we performed additional experiments in HEK293 cells. Plasmids carrying SpCas9/gRNA_AAVS1_/mKate2 and TdT were co‐transfected. After 72 h, we enriched for transfected cells using fluorescence‐activated cell sorting (FACS) (Figure 2f). We extracted the genomic DNA from the mKate2+ population and amplified the *AAVS1* locus via PCR (Supporting Material, Primers). The PCR products were then subjected to next‐generation amplicon sequencing in two batches (seq‐TdT_1‐4_ and seq‐TdT_5‐6_), each with their respective technical replicates. Sequencing results were analyzed based on our previously established pipeline.^[^ ^4^ ^]^ Briefly, the sequencing data from FASTQ files filtered with a regular expression‐based method to remove corrupted reads. The regular expression pattern targeted three key regions: a variable barcode region, a flexible indel region spanning 20 bp upstream and downstream of the cut site, and fixed regions matching the reference sequence. After filtration, wild‐type and substitution mutations were excluded to isolate the indel‐containing reads. For TdT‐mediated indels, the regular expression pattern was altered to accommodate insertions up to 10 bp in length. The filtered reads were further processed to extract indels, identify inserted or deleted sequences by alignment to the reference sequences, and generate indel frequency tables. With Cas9 alone, the top 20 indels consisted predominantly of deletions (Figure 2g), whereas co‐expression with pl‐TdT shifted the distribution entire toward insertions (Figure 2h). Moreover, we observed that the top 20 indels, which capture more than 30% of the total population, have consistent nucleotide content across all independent experiments. More specifically, 85% of the most frequent insertions contained nucleotides C, G, or both, consistent with previous findings that the two nucleotides flanking the cleavage site significantly influence indel distributions and nucleotide composition.^[^ ^21^, ^26^ ^]^

To mitigate noise from sequencing data, our data analysis pipeline incorporates strict regular expression filters to discard corrupted reads and to isolate short insertions and deletions. To explore whether any remaining noise could still interfere with our ability to distinguish between intra‐ and inter‐PUFs, we also performed a principal component analysis (PCA) on the observed indel compositions of the TdT‐PUF samples (Figure S5, Supporting Information). In this PCA space, the samples and their replicates (intra‐PUFs) cluster tightly together, whereas samples from different transfections (inter‐PUFs) remain well separated, suggesting that any remaining noise from sequencing is not expected to affect classification performance.

To quantify the similarity between indel frequencies in a population of cells resulting from individual transfections (i.e., inter‐PUF distance), we applied the pairwise Bray‐Curtis dissimilarity (BCD) metric ^[^ ^27^ ^]^ to the 12 samples, which include seq‐TdT_1‐6_ and their respective replicates (seq‐TdT_1r_ to TdT_6r_).^[^ ^4^ ^]^ We first used seq‐TdT_1_ as the reference and examined the indels of the top 5% to 100%, calculating the difference between intra‐PUF (defined as the variation between a specific PUF and its corresponding repeat or freeze‐thaw counterparts) and inter‐PUFs (defined as the variation between two different PUFs). We determined that the top 25% of indels provide good separation between intra‐PUF and inter‐PUFs (Figure S6, Supporting Information). We further calculated BCD between all sample pairs using the top 25% of indels and calculated the difference in fold change using each individual sample as the reference. The results show an average 3.408‐fold difference between intra‐ and inter‐PUFs, providing an acceptable classification (Figures S7 and S8, Supporting Information).

### 2.2 TdT‐Mediated Insertion Dependence on Time and Genomic Context

**Figure 3:**
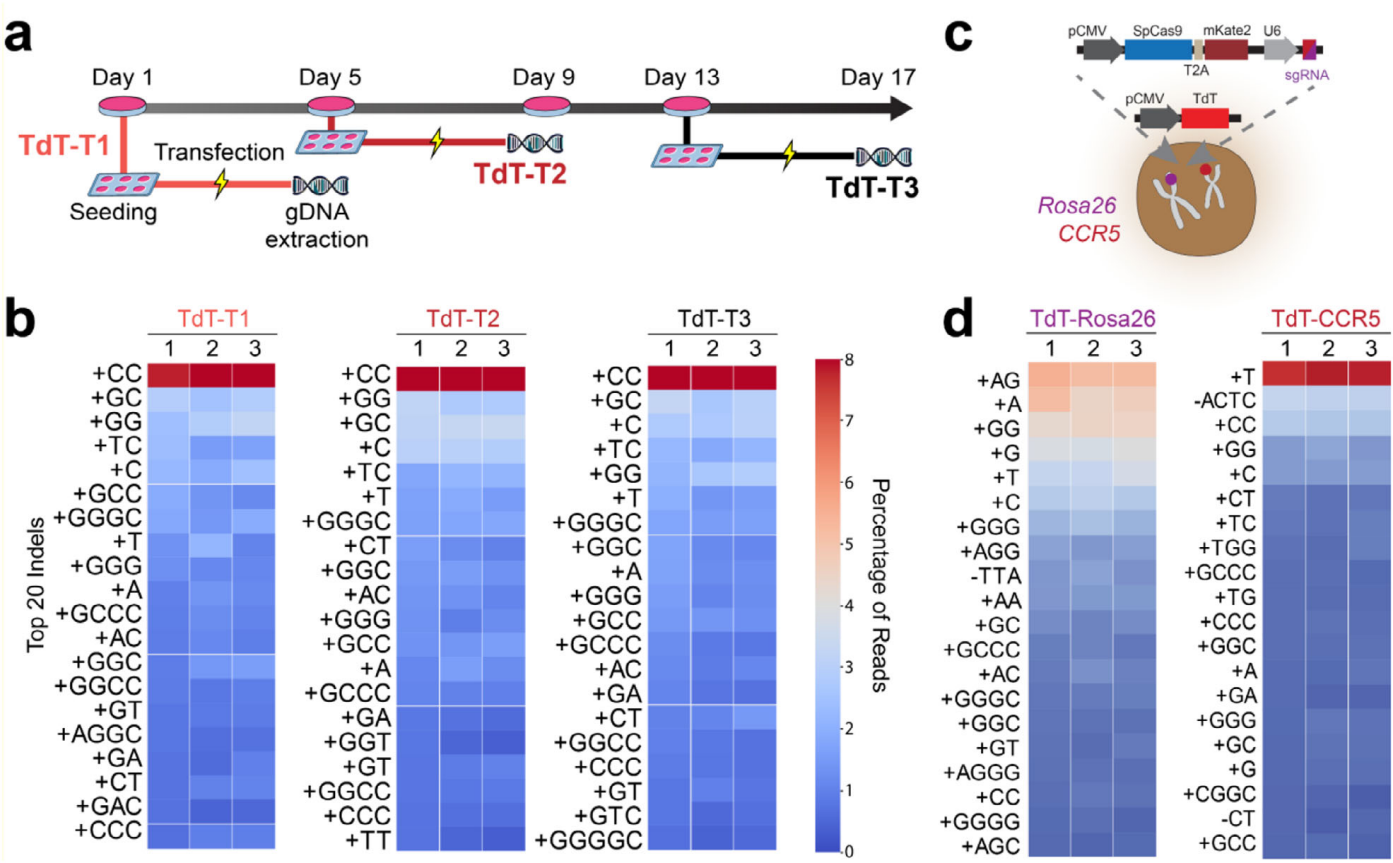
The relationship between TdT‐mediated indels and transfection time points and location. a) Illustration of the experimental timeline. The experiment was conducted at three time points: TdT‐T1, TdT‐T2, and TdT‐T3. For each time point, cells were seeded in a six‐well plate on the first day, transfections were performed on the second day, and cells were enriched via FACS on the fifth day (3 days post‐transfection), after which gDNAs were harvested. Cells were transfected at 3 time points (TdT‐T*n*), with 3 PUFs at each time point. b) Frequency plot of the top 20 indels at different transfection time points. c) Plasmid constructs used to target the *Rosa26* and the *CCR5* loci. d) Frequency plots of the top 20 indels at the hRosa26 and CCR5 sites.

To assess the possibility that transfections at different times could affect the TdT‐mediated indel distribution (e.g., due to differences in nucleotide availability and cell cycle stage), we conducted 3 independent transfections at 3 different time points using the same constructs as the previous experiment (**Figure** **3**a). For each timepoint, we selected mKate2+ cells via FACS 72 h post‐transfection and processed them using the same pipeline as in previous experiments. Across all time points, the most common insertion was two cysteines (CC), and the top 5 most common insertions were CC, C, GC, GG, and T (Figure 3b). BCDs were calculated pairwise using the top 25% of indels. (Figure S9, Supporting Information). The results show that the insertion distributions are not influenced by transfection time. We therefore conclude that the main determinant of insertion entropy and nucleotide distribution is the location of the editing (i.e., nucleotides that flank the cleavage site).

We also assessed the temporal stability of the TdT‐PUF design by generating an additional PUFs in HEK293 (seq‐TdT_S1_) cells using the same protocol and cultured it for 10 passages (approximately two days per passage). At each passage, we extracted genomic DNA and PCR‐amplified the edited regions as before. Using the transfected population as a reference (P0), we calculated the BCD across all 10 passages (Figure S10A, Supporting Information). When we compared this trend against the BCD to two other PUFs generated in parallel (seq‐TdT_S2_ and seq‐TdT_S3_), we found that the dissimilarity due to temporal instability remained below the lowest inter‐PUF distance, i.e., minimum identification threshold (Figure S10B, Supporting Information). These results demonstrate that our design remains stable for at least 10 passages or >20 days of continuous culturing. Notably, when compared to the first‐generation 2D barcode–indel PUFs reported by Li et al., which exceeded the minimum identification threshold by passage 6, the TdT‐PUF consistently maintained a lower dissimilarity metric throughout the time course (Figure S10C, Supporting Information).

To investigate whether different targeting loci and cleavage site nucleotide composition affect the TdT‐mediated indel pattern, we performed additional experiments with the *hRosa26* and *CCR5* safe harbor sites ^[^ ^28^, ^29^, ^30^ ^]^ of HEK293 cells (Figure 3c), following the same experimental protocol as the experiment targeting the *AAVS1* site. For each site, three transfections were performed: seq‐TdT_7‐9_ for the *hRosa26* site and seq‐TdT_10‐12_ for the *CCR5* site. We again observed cleavage site‐dependent insertion bias at both *hRosa26* (AT) and *CCR5* (CT) loci, highlighting the difference in indel compositions (Figure 3d). For these two sites, the frequencies of A and T insertions were higher than what was observed at the *AAVS1* site. For the *hRosa26* site, A was present in the two most dominant indels (Figure 3d, left), while for the *CCR5* site, T was the most dominant indel (Figure 3d, right).

To quantitatively assess the differences among sequences, we computed BCDs for all sequences at both the *hRosa26* and *CCR5* sites, using seq‐TdT_7_ and seq‐TdT_10_ as references. The results showed that using 25% of the indels can provide good separation between intra‐PUFs and inter‐PUFs (Figures S11 and S12, Supporting Information). Herein, pairwise BCDs were calculated for samples of seq‐TdT_7‐9_ and their repeats (seq‐TdT_7r‐9r_) and for samples of seq‐TdT_10‐12_ and their repeats (seq‐TdT_10r‐12r_). As was observed with the *AAVS1* locus, average differences between intra PUFs and inter PUFs were 2.810‐fold and 3.929‐fold for hRosa26 and CCR5 sites, respectively (Figures S13–S16, Supporting Information).

### 2.3 Enhancing PUF Classification by Removing Common Indels

For the results presented thus far, we quantified similarity between samples using a set of indel frequencies in a population of cells. We observed that simple post‐sequencing processing steps can improve classification performance. First, based on the analysis of indel compositions and frequencies among samples, we identified a set of indels appearing in most samples at high frequencies. Identical entries across all samples typically lead to increased similarity scores when comparing vectors, thereby resulting in reduced dissimilarity measures (e.g., Bray‐Curtis). In other words, while these common entries contribute to the overall similarity, they simultaneously reduce the discriminatory power of the comparison and our ability to robustly classify cell populations as we incorporate more PUFs.

**Figure 4:**
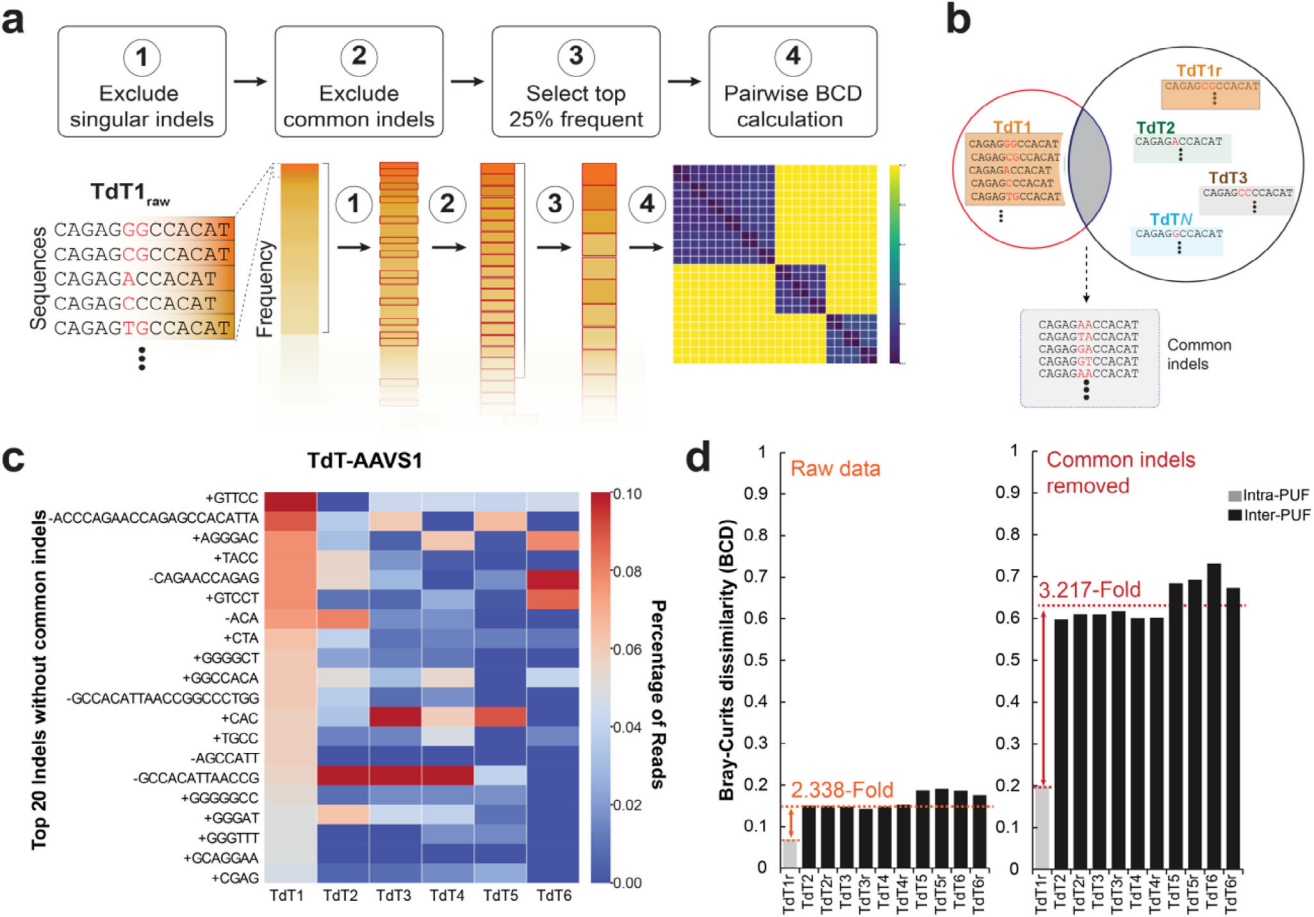
Exclusion of common indels for enhanced PUF classification. a) Overview of the computational processes, including exclusion of singular indel reads, removal of common indels, and use of the top 25% most frequent indels to perform pairwise BCD calculation. b) Schematic of the generation of common indels among the samples. c) Frequency plots of the top 20 indels from samples seq‐TdT_1‐6_ after common indel exclusion. d) BCD between seq‐TdT_1_ and the rest of the samples at the *AAVS1* locus after common indel removal. The fold changes shown are ratios between intra‐PUF BCD (gray) and the average of inter‐PUFs BCD (black).

To address this feature, we developed a post‐sequencing processing method which starts by omitting sequences that only appear a single time in each sample. Subsequently, we designate a set (list without duplicates) of indel sequences from a specific sample (e.g., seq‐TdT_1_), as the reference set and intersect it with every other sample (e.g., seq‐TdT_2‐6_) and their technical replicates (e.g., seq‐TdT_2r‐6r_). The intersection represents all common indels across samples, which we exclude from subsequent analysis. Lastly, the top 25% most abundant indels are used to compute pairwise BCD (**Figure** **4**a,b). The results show that removing common indels significantly impacts the differences in indel composition across various populations. This is evident in the indel frequency table, which initially featured many of the same sequences before filtering (Figure 2g), but now contains a heterogenous set of sequences (Figure 4c). For example, while the insertion GTTCC was the most dominant indel in seq‐TdT_1_, it was found to be absent in seq‐TdT_2_. Further, the majority of frequent indels observed in seq‐TdT_1_ were absent in seq‐TdT_6_ (Figure 4c). We proceeded with the pairwise BCD calculation using the top 25% most frequent indels. The results show that this procedure enhances the ability to distinguish between PUFed cell populations, increasing the average BCD fold change between intra‐PUF and inter‐PUFs from 2.338 to 3.217 when using seq‐TdT_1_ as reference (Figure 4d; Figure S17, Supporting Information). Applying this method to PUFs at different genomic loci, seq‐TdT_7‐9_ (hRosa26) and seq‐TdT_10‐12_ (CCR5), resulted in similar fold change increases: from 2.662‐fold to 4.349‐fold at hRosa26 and from 3.568‐fold to 6.161‐fold at CCR5 (Figures S18–S20, Supporting Information).

### 2.4 Classification of PUFs via Logistic Regression on TdT‐Mediated Indels

**Figure 5:**
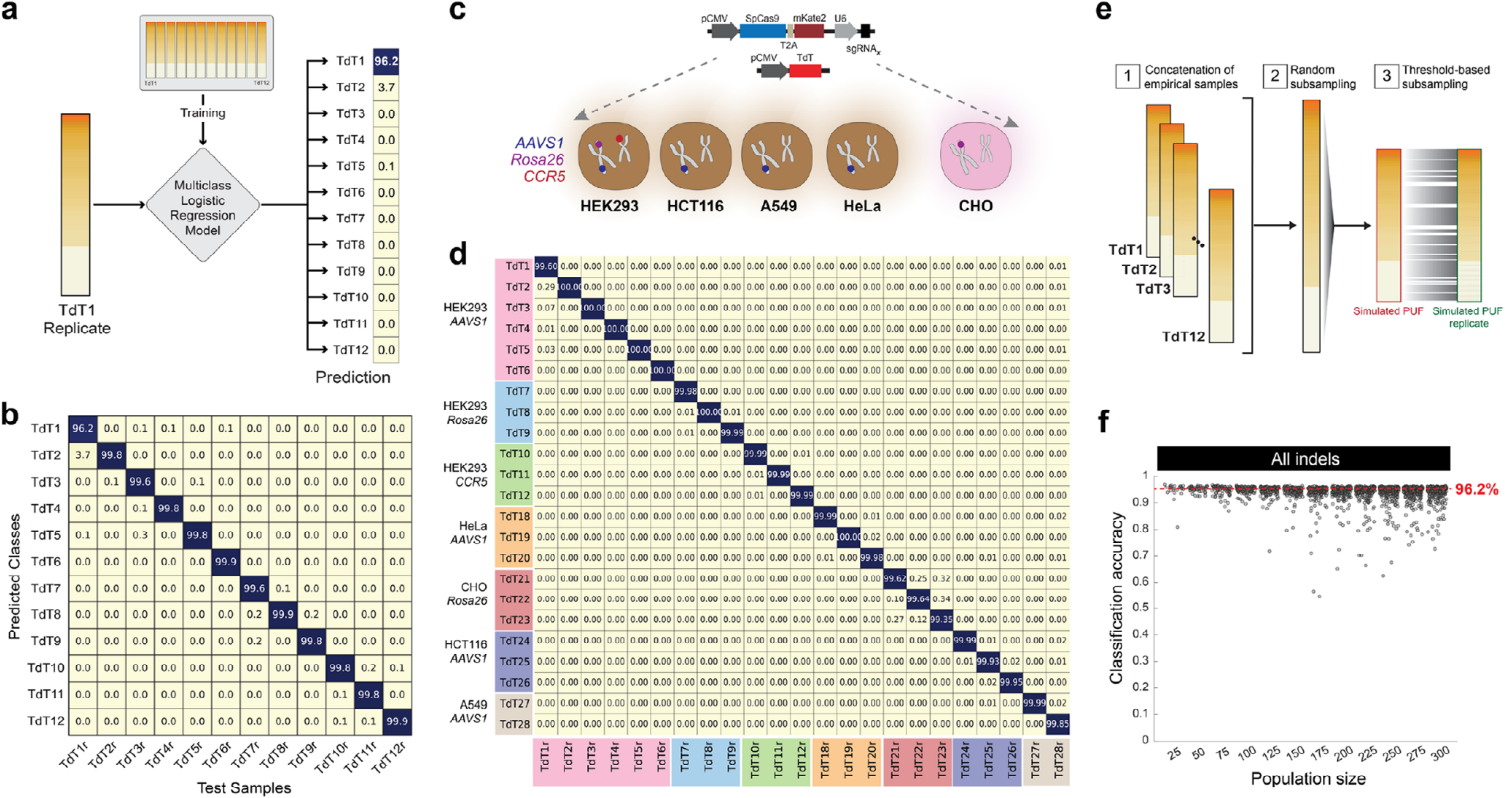
Multiclass logistic regression for classification of intra‐ and inter‐PUFs. a) The indel sequences and frequencies from the original samples (TdT_1_, TdT_2_…TdT*_12_*) were used as training data to build a logistic regression model. The technical replicates of these samples (e.g., TdT_1r_, TdT_2r_…TdT_12r_) were used as test data, and the trained model predicted the classes to which they belonged. b) Classification results of each test sample against every other sample as predicted by the logistic regression model trained in (a). c) Plasmid constructs used to target various loci in human cell lines (HEK293, HCT116, A549, HeLa) and Chinese Hamster Ovary cell line. The resulting PUFs created were used to train the multiclass logistic regression algorithm as described in (a). d) Classification results of each TdT‐PUF sample against every other sample as predicted by the logistic regression model trained in (c). e) To generate simulated PUFs, the sequences and corresponding frequencies of indels in every PUF were concatenated into a single comprehensive distribution of all observed PUFs. Simulated PUFs were then generated by randomly subsampling from this distribution. Technical replicates of these simulated PUFs were then created using random subsampling to reproduce the similarity levels measured experimentally. f) Classification accuracy of simulated PUFs generated by concatenating all indels.

While heuristic methods like the removal of common indels are sufficient for distinguishing between a small number of unique PUFs, we aimed to develop a more robust, data‐driven approach that efficiently identifies relevant features for provenance attestation in a large library of unique PUFs. To accomplish this, we employed a feature selection method commonly used in machine learning:^[^ ^31^ ^]^ multiclass logistic regression with an L1 penalty to eliminate less significant features while isolating features most informative for classification.^[^ ^32^, ^33^, ^34^, ^35^ ^]^ After training a logistic regression model on 12 PUFs generated across various loci, we evaluated its ability to classify the intra‐PUF that were included in the training set (e.g., seq‐TdT_1_ and its corresponding repeat seq‐TdT_1r_) (**Figure** **5**a). We observed that the model correctly classified the repeat with probabilities ranging from 96.2% to 99.9% (Figure 5b). This high level of accuracy indicates that the model can effectively distinguish between intra‐ and inter‐PUFs.

Next, we systematically evaluated the scalability (i.e., the number of PUFs that we can produce without risk of misidentification) of the feature selection method. First, we implemented TdT‐PUFs in alternative cellular contexts. We used the same experimental pipeline to construct PUFs in three additional human cell lines (HeLa, HCT116, A549) and Chinese hamster ovary (CHO) cells (Figure 5c). TdT‐mediated NHEJR was performed in the *AAVS1* locus using the same sgRNA target sequence, except for the CHO cell line, where we used the *Rosa26* locus. We then performed pairwise BCDs for all samples of the same cell lines and calculated the average differences between intra‐PUFs and inter‐PUFs (Figure S21, Supporting Information). The average distances are 4.006‐fold, 5.039‐fold, 5.627‐fold, and 1.661‐fold for HeLa, HCT116, A549, and CHO cell lines separately (Table S1, Supporting Information). Retraining the model on the expanded PUF library with L2 penalty retained classification accuracy at >99 % for every sample, demonstrating that supervised learning method scales effectively to an expanding set of PUF signatures (Figure 5d).

To introduce additional samples, we first concatenated all indel sequences and their respective frequencies from all experiments, creating a comprehensive probability distribution of observed indels. We then generated synthetic PUFs by randomly sampling (with replacement) from this distribution (Figure 5e). To create replicates of these simulated PUFs, we generated corresponding subsets with a 77% overlap in composition, reflecting the average overlap observed in experimentally derived PUFs (Figure S22, Supporting Information). We then trained logistic regression models for 25 to 300 simulated PUF samples and assessed the algorithm's ability to accurately discriminate between each intra‐ and inter‐PUF samples (Figure 5f). With the simulated PUFs, we observed a minimum average prediction accuracy above 95.2%. To identify the factor most affecting classification performance, we repeated the same procedure but limited the size of insertions in our simulated PUFs to a range of 3 to 7 base pairs (Figure S23, Supporting Information). Since TdT‐based indel formation contributes to entropy primarily through insertions, we reasoned that constraints on the size of insertions would hamper the ability to classify PUFs in large populations. As expected, reducing the indel size results in a significant number of samples with classification accuracy below 95%. Taken together, these simulations demonstrate how factors that contribute to the overall repair entropy (i.e., indel sequence identity, size, and frequency) impact PUF classification. Importantly, the results show that the TdT‐induced indel profiles yield sufficient entropy to afford robust classification of at least 100 PUFs (produced in a single batch) (Figure 5f).

## 3 Discussion

Cell line misidentification has been a persistent issue compromising the integrity of biomedical research since the 1960s.^[^ ^36^ ^]^ Despite advances in biotechnology and genome engineering, a reliable and practical standard for cell line authentication remains elusive, leading to significant waste of resources and potential misinterpretation of experimental results. In this manuscript, we present a novel solution to this critical problem by developing genetic PUFs that leverage the intrinsic entropy of DNA lesion repair mediated by TdT, a member of the X‐family of DNA polymerases. Our method exploits the natural variability introduced during TdT‐mediated repair, generating robust, unique, and virtually unclonable genetic identifiers directly within human cells. For fast and accurate authentication process, we implemented a machine learning‐assisted classifier that analyzes next‐generation sequencing data to precisely discriminate between individual genetic PUFs.

A key property of genetic PUFs is the inherent randomness arising from variations in the manufacturing protocol. Accordingly, we investigated the impact of transfection timing and genomic location selection for the PUF engineering. Our results show that the resulting indel nucleotide frequencies and distributions are independent of editing time. We also investigated the impact of TdT‐mediated indel generation across various genetic loci, each with a distinct cut site. Specifically, we evaluated 3 different sgRNAs with cleavage sites CG, AT, and CT; the results indicated unique indel outcomes at each of the target sites. Both parameters can be further explored to produce new generations of genetic PUFs. For example, considering that supplementing cultured cells with deoxyribonucleosides can alter TdT‐mediated indel outcomes,^[^ ^37^ ^]^ there is a direct opportunity to control the PUF entropy during manufacturing. Additionally, Cas variants with broadened compatibility for protospacer adjacent motifs (PAM)^[^ ^38^, ^39^ ^]^ can be utilized to target adjacent genome sites during the PUF engineering.

To demonstrate the versatility of TdT‐based PUFs, we applied the system across several commonly used immortalized cell lines, including HEK293, HCT116, HeLa, A549, and CHO. In these cell lines, TdT overexpression was well‐tolerated, with no detectable impact on cell viability or morphology. However, when applied to a primary fibroblast cell line (CRL‐2522), we observed both reduced editing efficiency and increased cytotoxicity upon co‐expression of Cas9 and TdT (Figure S24, Supporting Information). These results suggest that while TdT‐PUFs are broadly compatible with standard cell culture models, their implementation in primary or stem cells will require further optimization.

The proposed method offers significant improvements in the PUF manufacturing protocol (Figure S25, Supporting Information). By eliminating the need for barcoding prior to indel generation, we have reduced the production time from a few months to days. This change also decreases the total cost of engineering each PUF cell line by 3‐fold, including reagents, consumables, and NGS service. Despite these reductions in time and cost, it is important to note that the TdT‐based PUFs maintain similar levels of unclonability and robustness. Manual construction of a single Barcode‐Indel PUF requires establishing 500 individual cell lines and mixing them properly^4^, where the TdT PUF requires building 380 cell lines. Therefore, both methods are sufficiently secure to prevent unauthorized reproduction. Finally, incorporating logistic regression into our model ensures robust classification of TdT‐based PUFs.

To conclude, we demonstrate that TdT can be effectively harnessed to enhance intrinsic entropy during DNA lesion repair, enabling the rapid production of genetic PUFs that meet the criteria of robustness, uniqueness, and unclonability. Our results not only provide novel insights into the function of TdT but also, looking forward, represent a major advancement toward the practical application of genetic PUFs as a biosecurity primitive for cell line authentication and provenance verification, addressing a longstanding challenge in biomedical research.

## 4 Experimental Section

### Plasmid Cloning

The pCMV‐TdT plasmid was obtained from Addgene (126450). pCMV‐spCas9‐T2A‐mKate2‐U6‐sgRNA_AAVS1/sgRNA_CCR5/sgRNA_Rosa26 was cloned through standard molecular cloning. Briefly, oligos containing the desired spacer sequences were amplified using PCR and inserted into the vector plasmid using restriction enzyme digestions and ligated by T4 DNA ligase. Transformations were performed using NEB 5‐alpha Competent E. coli (NEB, C2987H). The plasmids were harvested and purified using the QIAprep Spin Miniprep Kit (Qiagen, 27 104).

### Cell Culture and Transfection

HEK293 cells were obtained from ATCC (CRL‐1573) and cultured in Dulbecco's Modified Eagle Medium – high glucose (Sigma–Aldrich, D5796), supplemented with 10% FBS, 1% Non‐Essential Amino Acids (Gibco, catalog. 11140076) and 1% Penicillin‐Streptomycin (Gibco, 15070063), at 37 °C and 5% CO_2_. 450000 cells were seeded into each well of 6‐well plates. After 24 h, 400 ng pCMV‐TdT plasmid and 1600 ng pCMV‐SpCas9‐T2A‐mKate2‐U6‐sgRNA_AAVS1/sgRNA_CCR5/sgRNA_Rosa26 plasmids (a total of 2000 ng DNA), were transfected into the cells with 4 µL JetPrime reagent (Polyplus, 101000046) in 200 µL buffer.

### Genomic DNA Extraction from Selected Cells

72 h post‐transfection, the media was removed and 500 µL 0.25% trypsin (Gibco, 25200114) was added to each well. After incubation at 37 °C for 5 min, 1.5 mL of complete media was added to each well to neutralize the trypsin. The cell suspension was then pelleted by centrifuge at 1500 rpm for 5 min. After removing the supernatant, the cell pellets were resuspended in 600 µL PBS (Corning, 21040CV). Subsequently, the cell suspensions were subjected to FACS to collect ≈200 000 cells expressing mKate2. The gDNAs were extracted using DNeasy Blood & Tissue Kits (Qiagen, 69504), following the standard protocol.

### NGS of the Targeted Region

The DNA fragments of the targeted regions were PCR‐amplified using ≈100 ng extracted gDNA as the template and primers listed in Table S2 (Supporting Information). The PCR conditions followed the standard protocol of Q5 Hot Start Master Mix (2X) (NEB, M0494S), 98 °C – 30 s, (98 °C –10 s, 63 °C –30 s, 72 °C –30 s) *35, 72 °C – 2 min, 4 °C –forever. PCR product sizes were examined by gel electrophoresis, with products of the correct size purified using HiBind DNA Mini Columns (Omega Bio‐TEK, DNACOL‐01). The purified amplicons were sent to Genewiz, Inc. for Amplicon‐EZ sequencing, including library preparation and 2×250 paired‐end sequencing on an Illumina MiSeq Sequencing System.

### Sequencing Data Analysis

Raw data were initially analyzed based on our previously established pipeline, modified to allow for TdT‐mediated insertions,^[^ ^4^ ^]^ as follows: 1) extract sequences from R1 fastq files; 2) filter out erroneous reads allowing deletions within 20 bp of the predicted cut site and insertions equal or smaller than 10 bp, with the remainder of the sequence matching the reference sequence; 3) extract indel sequences; 4) isolate the 40 bp containing the indel, including wild‐type sequences and substitutions; 5) identify insertions or deletions by aligning to reference sequences.

### Bray‐Curtis dissimilarity

Bray‐Curtis dissimilarities between sample *i* and sample *j* are calculated from the following equation after the samples are sorted into the same order:

(1) $$BC\;D_{ij}=\sum_{k=1}^{n}\frac{\left|n_{ik}-n_{jk}\right|}{n_{ik}+n_{ij}}$$

where n is the frequency of index k in each sample.

### Entropy Calculation

Shannon Entropies are calculated using the following equation:

(2) $$H\;\left(x\right)=\sum_{i=1}^{m}-p_{i}\log_{2}p_{i}$$

where i is the index of each unique indel, *p_i_* is the frequency of indel *i*, *m* is the total number of unique indels.

### Logistic Regression Model

A logistic regression model was trained using the Scikit‐learn library in Python, with “liblinear” as the solver for all models. First, all PUF indel profiles were preprocessed into a standardized format, with indel sequences and their normalized counts represented as feature vectors. The dataset was then divided into training set (data from original PUF samples) and a test set (data from replicate PUF samples). After training, the test dataset was used to examine the performance of the model. Both L1 (Lasso) and L2 (Ridge) regularization penalties were tested. Model performance was evaluated on the test set to determine classification accuracy. The full logistic regression training and evaluation script was available online.

### Apoptosis Assay

Apoptosis was measured using an Annexin V Alexa Fluor 488/Propidium Iodide (PI) staining kit (ThermoFisher, v13241) according to the manufacturer's protocol. Cells transfected with Cas9 or Cas9 + TdT expressing cassette were harvested, washed with cold PBS (Corning, 21040CV), and resuspended in 1X Annexin binding buffer at a final concentration of ≈1 × 10⁶ cells mL^−1^. For each 100 µL of cell suspension, 5 µL of Annexin V and 1 µL of 100 µg mL^−1^ PI solution were added, followed by incubation at room temperature for 15 min in the dark. Stained cells were analyzed using a flow cytometer using Annexin V with a 488‐nm laser, and 530/30 band‐pass filter, and PI with 561‐nm laser, and 610/20 band‐pass filter. The results were analyzed using FlowJo.

### Off‐Target Assay

Potential off‐target sites were predicted using Cas‐OFFinder, and primer pairs for each site were designed accordingly. Each off‐target region was PCR‐amplified using Q5 Hot Start High‐Fidelity 2X Master Mix (NEB, M0494S) under the following thermal cycling conditions: initial denaturation at 98 °C for 30 s; 35 cycles of 98 °C for 10 s, 63 °C for 30 s, and 72 °C for 30 s; followed by a final extension at 72 °C for 2 min and a hold at 4 °C. For heteroduplex formation, 5 µL of the unpurified PCR product was mixed with 2 µL of 10X NEBuffer 2 (NEB, B7002S) and 12 µL of nuclease‐free water, denatured at 95 °C for 5 min, and then gradually cooled to room temperature. Once cooled, 1 µL of T7 Endonuclease I (NEB, M0302S) was added to the reaction, followed by incubation at 37 °C for 15 min. Digestion products were analyzed on a 1% agarose gel to detect cleavage patterns indicative of indel formation at off‐target sites.

## Conflict of Interest

The authors declare that The University of Texas at Dallas has submitted a patent application relating to the genetic PUF engineering and authentication methods described in this manuscript.

## Supporting information



Supporting Information

Supporting Information

Supporting Information

Supporting Information

## Data Availability

The data that support the findings of this study are available from the corresponding author upon reasonable request.
